# Effects of Ryegrass and Exogenous Carbon Addition on Petroleum Hydrocarbon Removal and Soil Microbial Communities

**DOI:** 10.3390/molecules31213825

**Published:** 2026-10-23

**Authors:** Anyi Niu, Xuechao Sun, Ju Wang, Chunsheng Fang, Songjun Xu

**Affiliations:** 1 College of New Energy and Environment, Jilin University, Changchun 130012, China; 2 Key Laboratory of Groundwater Resources and Environment, Ministry of Education, Jilin University, Changchun 130021, China; 3 Jilin Province Key Laboratory of Water Resources and Environment, Jilin University, Changchun 130021, China; 4 School of Geography, South China Normal University, Guangzhou 510631, China

**Keywords:** petroleum-contaminated soil, ryegrass, microbial communities, plant-assisted remediation

## Abstract

Extensive oil contamination left by the Gulf War, together with arid and nutrient-poor conditions, poses persistent challenges to the bioremediation of petroleum-contaminated soils in Kuwait. This study examined the effects of ryegrass and different forms of exogenous carbon on petroleum hydrocarbon removal and soil microbial communities. Twelve pot treatments combined ryegrass planting with different doses of β-hydroxybutyric acid (HB) or polyhydroxybutyrate (PHB) in soil artificially contaminated with engine oil. Total petroleum hydrocarbons (TPH), organic acids, microbial communities, and predicted hydrocarbon-degradation functions were analyzed. At 150 d, ryegrass combined with PHB at 500 mg/pot produced the numerically highest mean TPH reduction (39.09%), exceeding that of the unplanted, unamended control by 18.91 percentage points. However, final TPH concentrations did not differ significantly from those with ryegrass alone or PHB alone at the same dose (500 mg/pot). Increasing the PHB dose reduced remediation performance. HB and PHB produced distinct patterns of organic acid change and significantly affected bacterial community composition. At 60 d, *Massilia* and *Dyadobacter* were relatively abundant in the ryegrass + PHB 500 mg/pot treatment. PICRUSt2 analysis of 16S rRNA gene sequences attributed predicted gene families associated with aromatic intermediate transformation to these genera. *Gordonia* was relatively abundant in the ryegrass + HB 550 mg/pot treatment at 150 d. These findings highlight taxa potentially relevant to hydrocarbon degradation, although their actual contributions require direct functional validation. The results indicate that the form and dose of exogenous carbon influence plant-assisted remediation and bacterial community responses under the tested conditions. Applicability to field-contaminated soils in Kuwait requires further validation.

## 1. Introduction

Large-scale crude oil spills during the 1990–1991 Gulf War created numerous oil lakes and contaminated soil piles in Kuwait [1]. Remediation projects addressing this legacy contamination cover approximately 114 km^2^ of oilfield land [2]. Some petroleum constituents remain in contaminated soils after decades of weathering [3,4]. The extensive area affected makes soil transport and energy and material requirements major constraints on the choice of remediation strategy, highlighting the practical value of in situ microbial remediation. A systematic review of oily-sludge treatment research has highlighted the limited consideration of cost-effectiveness in existing treatment approaches [5]. Kuwait has a hot, dry climate and nutrient-poor soils, where limited water and nutrient availability can constrain microbial hydrocarbon degradation. Local trials have shown that irrigation combined with mixing improves oil removal and that compost promotes petroleum hydrocarbon degradation in contaminated Burgan oilfield soils through biostimulation [6,7]. Improving the soil environment for microorganisms is therefore an important means of increasing remediation efficiency [8].

Plants modify local soil conditions through root activity and influence the microorganisms involved in petroleum hydrocarbon transformation. Field surveys of contaminated sites in Kuwait have identified native plants capable of growing in petroleum-contaminated habitats [9]. In a ryegrass soil-column experiment, aliphatic hydrocarbons disappeared more rapidly from planted soil, accompanied by increased microbial abundance and respiration [10]. Hydrocarbon degradation varies among combinations of ryegrass and microbial strains, while nutrient supply also affects the abundance and expression of hydrocarbon-degradation genes in the ryegrass rhizosphere [11,12]. Diesel stress can also alter the composition of ryegrass root metabolites, some of which attract specific bacteria [13].

In addition to root-derived inputs, biodegradable polymers can supply carbon to some soil microorganisms. Microbial extracellular enzymes can depolymerize poly(3-hydroxybutyrate) (PHB), releasing low-molecular-weight products, including β-hydroxybutyric acid (HB) [14]. Particles of PHB-related copolymers can create localized zones of rapid microbial growth and elevated extracellular enzyme activity [15]. PHB addition also alters soil respiration, bacterial and fungal communities, and nutrient transformations [16,17]. However, some PHB experiments have also reported reduced plant biomass, with responses depending on soil sand content, co-applied amendments, and inoculation conditions [16,18,19]. In an incubation of unplanted agricultural soil, biodegradable microplastics, including PHB, slowed the natural attenuation of polycyclic aromatic hydrocarbons [20]. Microbial utilization of an amendment therefore does not necessarily improve petroleum hydrocarbon removal.

Root-derived inputs also modify the carbon environment available to microorganisms during plant-assisted remediation. The composition of root exudates and the stage of incubation can influence the expression of hydrocarbon-degradation genes [21]. Stable isotope tracing has directly linked specific microorganisms in the ryegrass rhizosphere to benzo[a]pyrene utilization [22]. Root inputs may therefore alter microbial responses to HB or PHB addition, and amendment effects observed in unplanted soil may not translate directly to planted soil.

Specifically, we hypothesized that ryegrass cultivation would modify soil microbial community responses to HB and PHB addition. We further hypothesized that increasing the dose of either amendment would not consistently improve TPH removal within the tested dose range. Based on preliminary screening of candidate plants, ryegrass was selected for a pot experiment using soil artificially contaminated with engine oil. A fully crossed design combined the presence or absence of ryegrass with six amendment conditions: no amendment, three HB doses, and two PHB doses. Remediation performance was evaluated using residual total petroleum hydrocarbons (TPH) at 60 and 150 d and the decline between the two sampling times. The effects of ryegrass planting and the additional effects of HB or PHB under each planting condition were compared. Soil properties, bacterial and fungal communities, and predicted bacterial hydrocarbon-degradation functions were examined to identify microbial responses associated with differences in TPH. Organic acids measured in planted treatments were used to support interpretation of the soil carbon environment. The study aimed to identify treatments with favorable TPH removal and candidate taxa associated with lower residual TPH for subsequent culture-based and hydrocarbon-degradation assays. This study provides preliminary evidence for evaluating plant-assisted microbial remediation at a TPH loading informed by published field measurements from Kuwait. Its applicability to field-contaminated Kuwaiti soils remains to be established.

## 2. Results

### 2.1. Changes in TPH and C10–C40 Under Different Treatments

**Figure 1:**
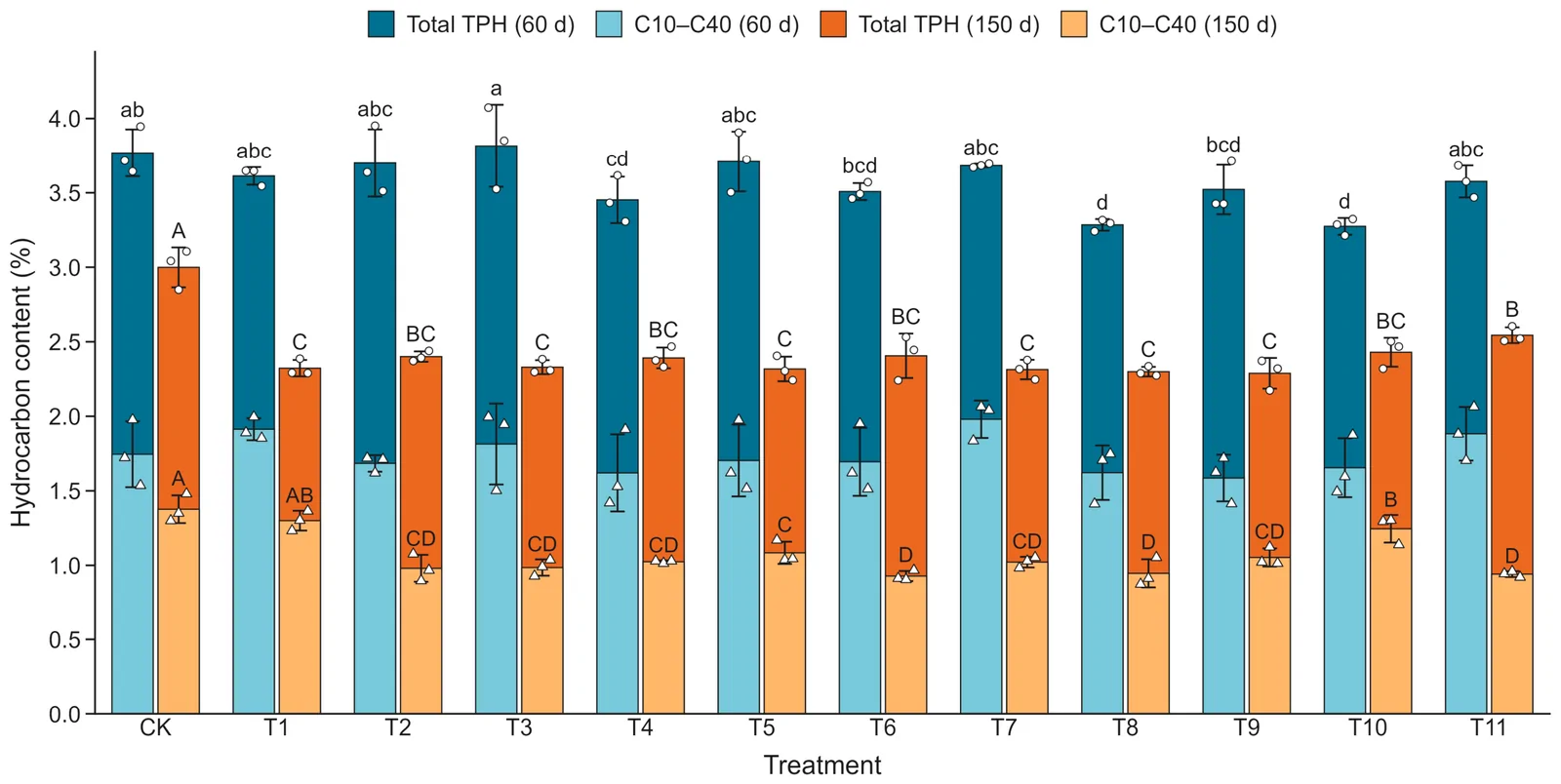
Changes in total TPH and its C10–C40 fraction under different treatments. Two bars per treatment represent 60 and 150 d. The full height of each bar represents mean total TPH, and the lighter area within the same bar indicates mean C10–C40 content. Bars and error bars show means ± SD. White circles and triangles represent individual-pot values for total TPH and C10–C40, respectively (*n* = 3). Lowercase letters indicate comparisons at 60 d and uppercase letters indicate comparisons at 150 d. Letters above the full bars refer to total TPH, whereas letters within the bars refer to C10–C40. C10–C40 is a component of total TPH and is used only to describe composition; remediation performance is evaluated solely by total TPH reduction. Within each measurement and sampling time, treatments sharing no letter differ significantly (*p* < 0.05; Duncan’s multiple range test).

The day-0 TPH content after artificial contamination was 3.757 ± 0.018%. During the later remediation period (60–150 d), TPH declined further in every treatment, with decreases of 0.770–1.486 percentage points. The ryegrass + HB 150, 350, and 550 mg/pot treatments showed relatively large declines during this interval, at 1.486, 1.393, and 1.370 percentage points, respectively. Across the full remediation period (0–150 d), however, ryegrass + PHB 500 mg/pot produced the largest TPH reduction. At 150 d, TPH in this treatment was 2.289 ± 0.103%, representing a 39.09% reduction from the initial value. In comparison, TPH in the unplanted, unamended treatment was 2.999 ± 0.134%, a reduction of 20.18%. The difference in reduction was 18.91 percentage points, indicating that ryegrass + PHB 500 mg/pot produced the numerically largest TPH decline (Figure 1).

TPH reductions with ryegrass alone and PHB 500 mg/pot without ryegrass were 38.18% and 38.80%, respectively, only 0.91 and 0.29 percentage points below that with ryegrass + PHB 500 mg/pot. Final TPH contents in these two treatments did not differ significantly from that in the combined treatment (*p* = 0.6631 and *p* = 0.8774, respectively). Supplementary model-based estimates of the corresponding differences in residual TPH, together with their 95% confidence intervals, are presented in Table S6. Increasing the PHB dose to 1500 mg/pot in planted soil resulted in a final TPH content of 2.544 ± 0.053% and a reduction of 32.29%, which was 6.80 percentage points lower than the reduction at 500 mg/pot. Final TPH was significantly higher at the larger dose (*p* = 0.0037). Final TPH contents in the ryegrass + HB 150, 350, and 550 mg/pot treatments ranged from 2.314% to 2.330%, showing no sustained decline with increasing HB dose.

At 60 d, planting and amendment condition had significant main effects on residual TPH, whereas their interaction was not significant (Table S5). Averaged across amendment conditions, residual TPH was 0.156 percentage points higher in planted than in unplanted pots. At 150 d, both main effects and their interaction were significant.

C10–C40 contents did not differ significantly among treatments at 60 d (*p* = 0.2852), but treatment differences were significant at 150 d (*p* < 0.0001). The unplanted + HB 550 mg/pot treatment had the lowest final C10–C40 content, at 0.928 ± 0.034%, but did not have the lowest TPH content. Thus, treatment rankings based on C10–C40 did not fully correspond to those based on residual TPH. TPH reduction was therefore used as the primary measure of overall remediation performance, while C10–C40 characterized changes in hydrocarbon composition. Shoot, root, and total dry biomass of ryegrass at 150 d are summarized in Table S4.

### 2.2. Distinct Organic Acid Dynamics Under HB and PHB Addition

All 65 organic acid assay entries were assigned to six chemical structural classes. Aliphatic and alicyclic carboxylic acids accounted for 27 entries, and benzenoid carboxylic acids and their derivatives accounted for 19; together, these classes represented 70.8% of all assay entries (Figure S1). During the later remediation period (60–150 d), 12 organic acid entries declined in all six planted treatments, 9 increased in all treatments, and 26 showed treatment-dependent directions of change. (R)-3-Hydroxybutyric acid, L-malic acid, and levulinic acid declined in every treatment, whereas α-ketoglutaric acid and sebacic acid increased. Suberic and azelaic acids showed pronounced treatment differences: both increased only in the ryegrass + PHB 1500 mg/pot treatment and declined in the other five treatments. These seven organic acids were selected for detailed analysis based on their relevance to the amendments, their potential relevance to petroleum hydrocarbon transformation, and their treatment-dependent patterns. The remaining entries are shown in the complete heatmap (Figure S1).

**Figure 2:**
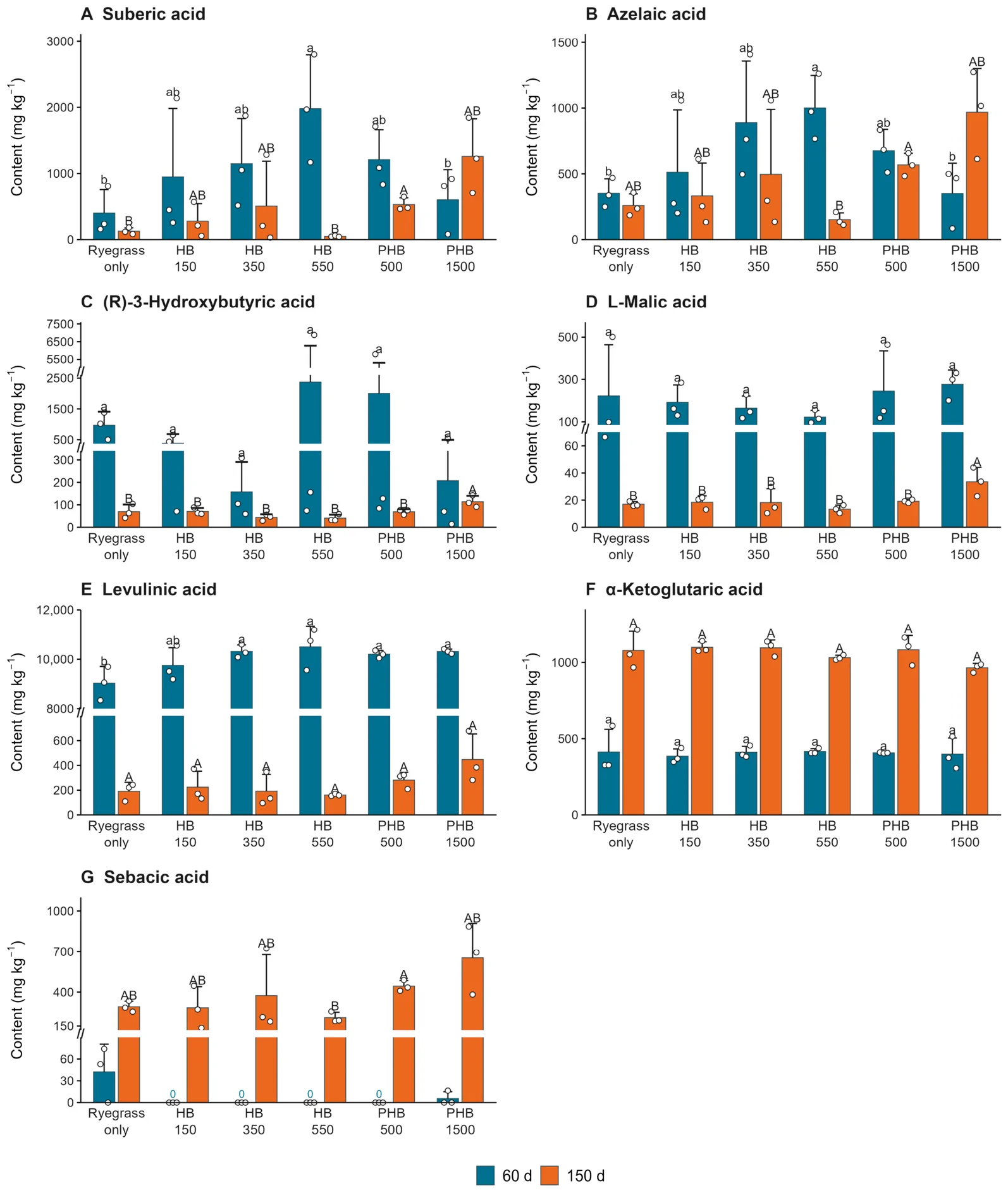
Changes in the concentrations of seven focal organic acids under different treatments. Axis segments in panels with broken y-axes use different scales; breaks are indicated on the axes. Bars represent means, error bars represent SD, and white points represent individual-pot measurements. Lowercase letters indicate comparisons at 60 d and uppercase letters indicate comparisons at 150 d. Blue and orange bars denote 60 and 150 d, respectively. Within each compound and sampling time, treatments sharing no letter differ significantly (*p* < 0.05), using the comparison procedure specified in Section 4.6.

Among the selected organic acids, suberic and azelaic acids showed particularly distinct directions of change among treatments. From 60 to 150 d, their concentrations declined by 97.5% and 84.8%, respectively, in the ryegrass + HB 550 mg/pot treatment, but increased by 108.6% and 176.1% in the ryegrass + PHB 1500 mg/pot treatment (Figure 2A,B). At 150 d, suberic, azelaic, and sebacic acid concentrations in ryegrass + PHB 500 mg/pot were 10.6, 3.7, and 2.1 times those in ryegrass + HB 550 mg/pot, respectively. The treatment differences were significant for all three compounds (Games–Howell, *p* < 0.05; Figure 2A,B,G).

At 150 d, (R)-3-hydroxybutyric acid and L-malic acid concentrations in the ryegrass + PHB 1500 mg/pot treatment were 114.08 ± 26.17 and 33.53 ± 10.56 mg/kg, respectively, significantly higher than in the other five treatments (Duncan, *p* < 0.05; Figure 2C,D). Nevertheless, these concentrations were 45.0% and 87.9% lower, respectively, than at 60 d in the same treatment. Levulinic acid declined markedly in all six treatments, by 95.7–98.5%. At 150 d, neither levulinic acid nor α-ketoglutaric acid differed significantly among treatments (*p* > 0.05; Figure 2E,F).

### 2.3. Differential Responses of Bacterial and Fungal Communities to Planting and Carbon Amendments

**Figure 3:**
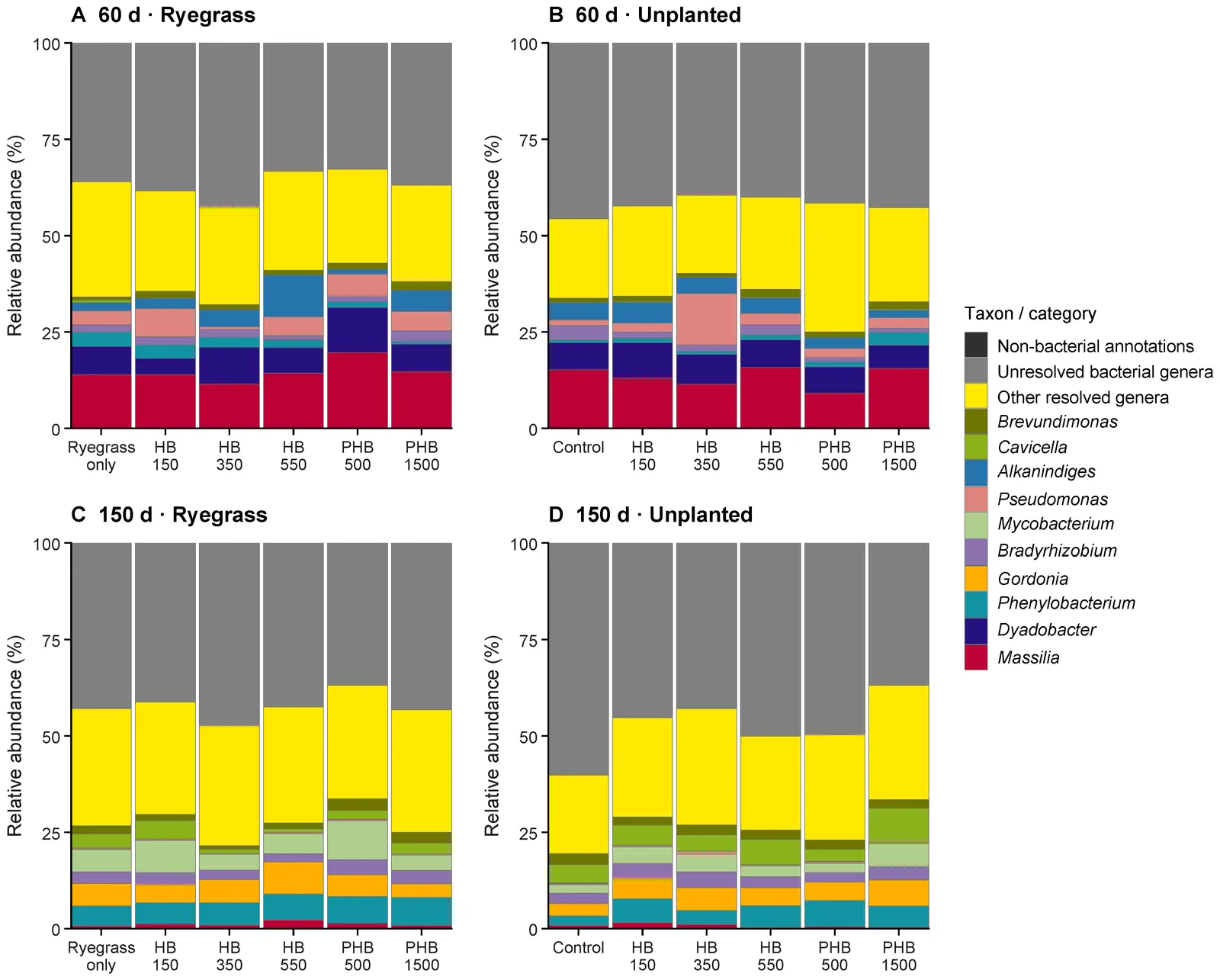
Bacterial genus composition at different sampling times and under different planting conditions. Each bar represents the equally weighted mean relative abundances of three pots in the same treatment. The 10 taxonomically assigned bacterial genera were selected consistently based on their mean relative abundances across all 72 samples. Other bacterial genera, bacteria unresolved at the genus level, and non-bacterial sequences remain in the denominator used to calculate relative abundance. Differences among bars are descriptive only.

The dominant bacterial genera differed between 60 and 150 d. At 60 d, the three taxonomically assigned genera with the highest mean relative abundances across all 36 pots were *Massilia* (14.03%), *Dyadobacter* (7.55%), and *Pseudomonas* (4.35%) (Figure 3). In the ryegrass + PHB 500 mg/pot treatment, mean relative abundances of *Massilia* and *Dyadobacter* were 19.61% and 11.77%, respectively, exceeding those with ryegrass alone by 5.69 and 4.44 percentage points. *Massilia* accounted for 9.14% in the unplanted + PHB 500 mg/pot treatment, 6.04 percentage points below the unplanted, unamended treatment. At 150 d, the three genera with the highest mean relative abundances were *Phenylobacterium* (5.58%), *Gordonia* (5.40%), and *Mycobacterium* (4.97%) (Figure 3). *Gordonia* accounted for 8.27% in ryegrass + HB 550 mg/pot, 2.44 percentage points above ryegrass alone. *Mycobacterium* accounted for 10.01% in ryegrass + PHB 500 mg/pot, 4.25 percentage points above ryegrass alone. *Cavicella* accounted for 8.92% in unplanted + PHB 1500 mg/pot, 4.23 percentage points above the unplanted, unamended treatment. Among planted treatments, mean relative abundances of *Paracoccus* were 4.83%, 5.06%, and 2.57% with ryegrass alone, ryegrass + HB 550 mg/pot, and ryegrass + PHB 1500 mg/pot, respectively. Corresponding abundances were 5.83%, 8.27%, and 3.56% for *Gordonia* and 0.56%, 0.22%, and 1.43% for the fungal genus *Penicillium* (Table S3).

In the exploratory genus-level analysis, three taxonomically assigned bacterial genera remained significant after BH correction across all estimable treatment comparisons and passed the pseudocount sensitivity check for zero handling. All of these differences occurred in unplanted soil (Figure S2). At 60 d, ANCOM-BC2 bias-corrected abundances of *Cnuella* were lower in the unplanted + HB 350 mg/pot and unplanted + PHB 500 mg/pot treatments than in the unplanted, unamended treatment (both q = 0.020). At 150 d, corrected abundances of *Citrifermentans* were lower in the unplanted + HB 350 mg/pot, HB 550 mg/pot, and PHB 500 mg/pot treatments than in the unplanted, unamended treatment (q = 0.0011–0.020). Here and throughout, q denotes a *p* value adjusted for multiple comparisons using the Benjamini–Hochberg procedure. Corrected abundance of *Rhodopseudomonas* was higher in the unplanted + HB 550 mg/pot treatment than in the unplanted, unamended treatment (q = 0.020). No bacterial genera in planted soil met both the significance and sensitivity-check criteria.

**Figure 4:**
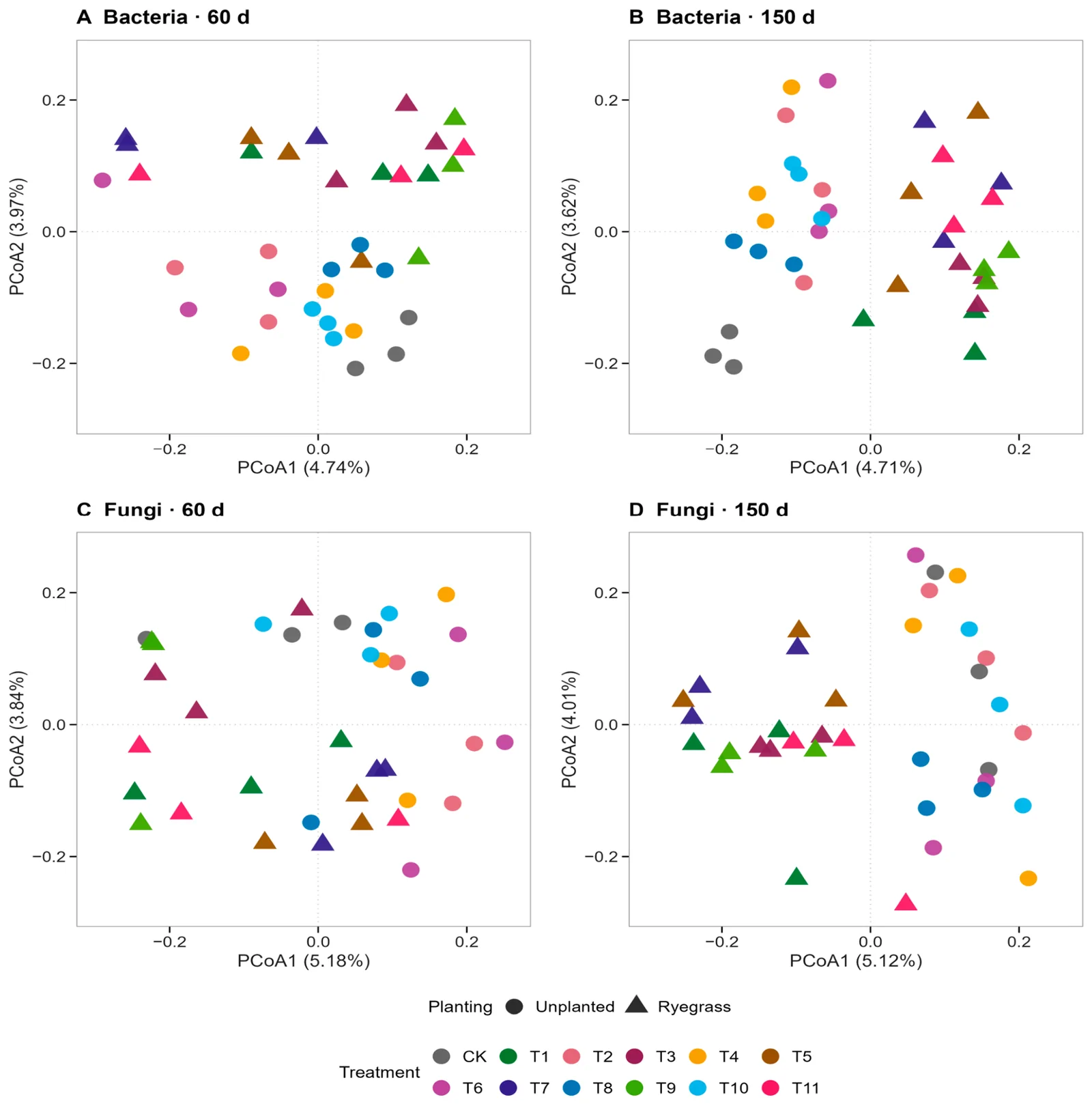
PCoA of bacterial and fungal communities at different sampling times. Panels (**A**) and (**B**) show bacteria at 60 and 150 d, and panels (**C**) and (**D**) show fungi at 60 and 150 d, respectively. Each panel includes samples from 18 planted and 18 unplanted pots. Colors distinguish CK and T1–T11; circles and triangles indicate unplanted and planted pots, respectively. CK is the unplanted, unamended treatment, and T1 is ryegrass alone. T2/T3, T4/T5, T6/T7, T8/T9, and T10/T11 represent unplanted/planted treatments receiving HB 150, HB 350, HB 550, PHB 500, and PHB 1500 mg/pot, respectively. Each panel was independently ordinated using the complete ASV matrix. Axis percentages indicate the variation explained within that panel. Community differences are evaluated using PERMANOVA on the complete distance matrices. Coordinates in different panels cannot be used to infer movement of samples over time.

PCoA showed separation between planted and unplanted samples at both 60 and 150 d (Figure 4). Amendment treatments were more clearly differentiated in bacterial communities, whereas fungal samples overlapped more extensively. PERMANOVA further tested community differences between planted and unplanted treatments. Bacterial communities differed significantly at both 60 and 150 d, with consistent results from Euclidean distances after Hellinger transformation and from Bray–Curtis distances (all q = 0.0006). Fungal communities also differed at 60 d (Hellinger q = 0.0054; Bray–Curtis q = 0.0045). At 150 d, however, the fungal planting effect was significant only in the Hellinger analysis (q = 0.0127), not in the Bray–Curtis analysis (q = 0.1214). The effect of planting on bacterial communities was therefore consistent at both sampling times, whereas the fungal result at 150 d depended on the distance metric.

When individual amendment doses were analyzed, significant effects on bacterial communities occurred mainly at 150 d. With no amendment, the three HB doses, and the two PHB doses treated as six separate levels, both the amendment main effect and its interaction with planting were significant at 150 d in the Hellinger and Bray–Curtis analyses. In the Hellinger analysis, amendment and the amendment × planting interaction explained 21.1% and 19.8% of community variation, respectively, after controlling for the other model terms (q = 0.0024 and 0.0174). When the three HB doses were pooled into an HB category and the two PHB doses into a PHB category, overall differences among no amendment, HB, and PHB were significant for bacteria under both planting conditions at both sampling times (R^2^ = 0.124–0.133, q ≤ 0.0144). In unplanted soil, all pairwise comparisons among the three categories were significant at 60 d (q = 0.020–0.026), and both HB and PHB differed from no amendment at 150 d (q = 0.020 and 0.026, respectively). In planted soil, no pairwise comparison remained significant after BH correction at 60 d; at 150 d, only HB and PHB differed significantly (q = 0.020). Neither the amendment main effect nor the planting × amendment interaction was significant for fungal communities at either sampling time (q ≥ 0.0566).

### 2.4. Treatment-Related Variation in Predicted Hydrocarbon-Degradation Functions

The PICRUSt2 prediction tables contained 7766 KOs at 60 d and 7788 at 150 d. PCA was performed separately for each sampling time and planting status, and the first two axes jointly explained 52.79–67.11% of the variation in predicted functional composition (Figure S3). After doses were pooled into no amendment, HB, and PHB categories, PERMANOVA was conducted in four subsets: planted soil at 60 d, unplanted soil at 60 d, planted soil at 150 d, and unplanted soil at 150 d. Amendment category explained 10.41%, 8.44%, 14.52%, and 23.18% of overall functional variation, respectively, with unadjusted *p* values of 0.5982, 0.7318, 0.2372, and 0.0416. After joint BH correction of the four tests, the corresponding q values were 0.7318, 0.7318, 0.4744, and 0.1664; none was significant. Although all four amendment-category tests for bacterial community composition remained significant after correction, treatment differences in overall predicted function lacked corresponding adjusted statistical support.

**Figure 5:**
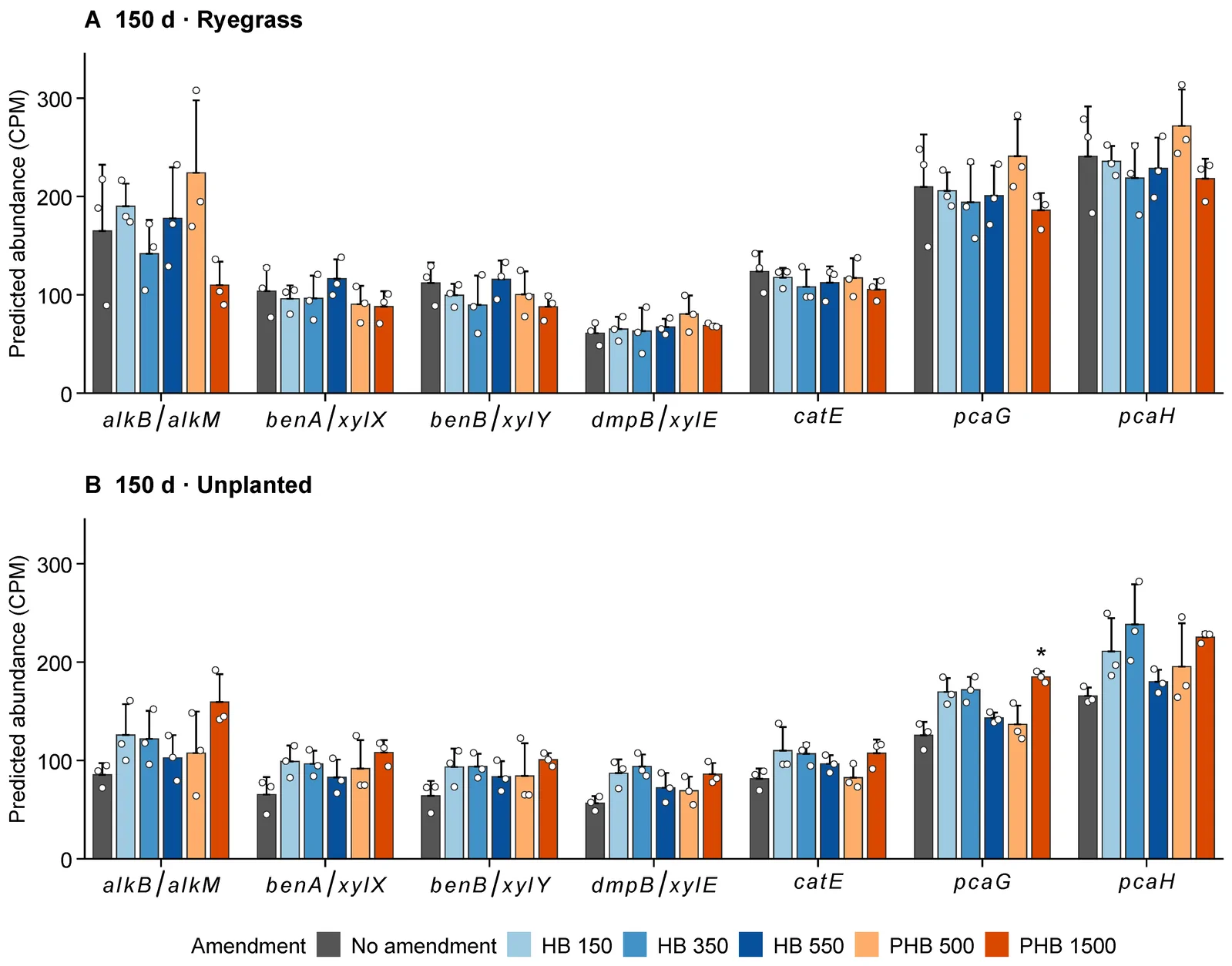
Seven predicted petroleum hydrocarbon degradation functions in planted and unplanted soil at 150 d. Upper panel (**A**) and lower panel (**B**) show predicted abundances in planted and unplanted soil, respectively. All seven prespecified focal KOs are displayed: *alkB*/*alkM* (K00496), *benA*/*xylX* (K05549), *benB*/*xylY* (K05550), *dmpB*/*xylE* (K00446), *catE* (K07104), *pcaG* (K00448), and *pcaH* (K00449). Both panels use the same y-axis range. Bars represent the means of three independent pots, error bars extend from the mean to mean + SD, and open circles represent individual-pot values. CPM was calculated by normalizing to 10^6^ using all supplied KO predictions in each pot as the denominator. An asterisk indicates a difference from the unamended control at the same sampling time and planting status that remained significant after BH correction in the original complete comparison family (q < 0.05). The only asterisk is for *pcaG* in the unplanted + PHB 1500 mg/pot treatment in panel (**B**). Colors indicate amendment conditions, and numbers following HB/PHB indicate mg/pot. No amendment denotes ryegrass alone in (**A**) and the unplanted, unamended treatment in (**B**).

Seven KOs directly associated with petroleum hydrocarbon transformation were examined in greater detail. These covered initial alkane oxidation (*alkB*/*alkM*), benzoate activation (*benA*/*xylX* and *benB*/*xylY*), catechol ring cleavage (*dmpB*/*xylE* and *catE*), and protocatechuate ring cleavage (*pcaG* and *pcaH*) (Figure 5). Among the six planted treatments at 150 d, ryegrass + PHB 500 mg/pot had the highest mean predicted abundances of *alkB*/*alkM*, *pcaG*, and *pcaH*, at 224.09, 240.91, and 271.75 CPM, respectively. Corresponding values with ryegrass alone were 165.02, 209.80, and 240.73 CPM. None of these three differences from ryegrass alone remained significant after BH correction (q = 0.555–0.678).

Of the 140 prespecified comparisons across the seven KOs, only *pcaG* in the unplanted + PHB 1500 mg/pot treatment at 150 d remained significant after BH correction. Its predicted abundance was 184.93 ± 5.72 CPM, 47.21% higher than the 125.63 ± 13.51 CPM in the unplanted, unamended treatment (q = 0.0287; Figure 5B). The other subunit of the same enzyme complex, *pcaH*, did not remain significant after correction (q = 0.1737), and no significant differences were detected for the other focal KOs. This significant result occurred in unplanted soil receiving PHB at 1500 mg/pot, whereas ryegrass + PHB 500 mg/pot produced the largest TPH reduction. An increase in a single predicted KO therefore did not correspond to better remediation performance.

## 3. Discussion

### 3.1. TPH Removal Under Ryegrass Cultivation and PHB Addition

When TPH reduction was used as the sole criterion for remediation performance, ryegrass + PHB 500 mg/pot had a TPH content of 2.289 ± 0.103% at 150 d, a 39.09% decrease from the common initial value and the numerically highest mean reduction among the tested treatments. The unplanted, unamended treatment showed a reduction of only 20.18%, giving a difference of 18.91 percentage points. Thus, ryegrass combined with PHB at 500 mg/pot produced the numerically highest mean TPH reduction under the tested conditions. The reported percentage reductions are estimates relative to the shared day-0 baseline, rather than pot-specific changes from individually measured starting concentrations. Any remaining variation in initial TPH among pots, or uncertainty in the common baseline, could affect these estimates.

Matched comparisons between planted and unplanted treatments show that the benefit of ryegrass was most evident without exogenous carbon addition. Ryegrass increased TPH reduction from 20.18% to 38.18%, an increase of 18.00 percentage points, with significantly lower final TPH concentrations in planted soil. This plant-associated enhancement is consistent with rhizosphere stimulation of microbial hydrocarbon degradation. In diesel-contaminated soil, Kaimi et al. [23] reported lower residual diesel concentrations together with higher aerobic bacterial abundance and dehydrogenase activity in the ryegrass rhizosphere than in root-free soil, providing a mechanistic basis for this interpretation.

In contrast, final TPH concentrations did not differ significantly between planted and unplanted treatments receiving the same HB or PHB dose. Thus, the substantial improvement associated with ryegrass alone was not accompanied by a significant additional planting benefit under carbon-amended conditions. Carbon-source characteristics also provide context for interpreting the treatment responses. Unlike readily available HB, PHB must first undergo microbial depolymerization into low-molecular-weight products and may therefore provide a slower, sustained carbon input. PHB has been used as a slow-release carbon source in bioremediation [24].

The bacterial community and functional prediction results provide further context for the response to PHB addition in planted soil. At 60 d, relative abundances of *Massilia* and *Dyadobacter* in ryegrass + PHB 500 mg/pot were 19.61% and 11.77%, respectively, exceeding those with ryegrass alone by 5.69 and 4.44 percentage points. These genus-level patterns are interpreted as exploratory, as no bacterial genera in planted soil met both the differential-abundance significance and sensitivity-check criteria. Taxonomic attribution of predicted functions further indicated that *Massilia* accounted for 41.23% of predicted *dmpB*/*xylE* associated with catechol ring cleavage, while *Dyadobacter* accounted for 46.99% of predicted *catE*. *Massilia* also accounted for a proportion of the predicted abundances of *pcaG* and *pcaH*. A plausible interpretation of these taxonomic patterns and functional predictions is that *Massilia* and *Dyadobacter* may participate in the transformation of aromatic petroleum hydrocarbon intermediates in the ryegrass + PHB 500 mg/pot treatment. At 150 d, this treatment also had the highest mean predicted abundances of *alkB/alkM*, *pcaG*, and *pcaH* among the six planted treatments. These numerical trends suggest possible variation in predicted hydrocarbon-transformation potential. However, the differences from ryegrass alone were not significant after BH correction (q = 0.555–0.678). These predictions may inform further investigation of potential microbial roles in petroleum hydrocarbon transformation. The contributions of individual taxa to TPH removal remain to be evaluated through targeted functional studies.

The benefit of PHB did not increase with dose. TPH reduction with ryegrass + PHB 1500 mg/pot was 32.29%, 6.80 percentage points below that at 500 mg/pot, and final TPH was significantly higher at the larger dose (*p* = 0.0037). Studies of PHBV in soil have shown that biodegradable polymers can create hotspots of carbon and nutrient turnover and restructure bacterial communities [15]. Higher additions can also reduce plant nitrogen status and microbial activity, producing dose-dependent adverse effects [25]. These observations suggest that PHB at 500 mg/pot may have supplied carbon that helped sustain hydrocarbon-degrading microorganisms, whereas 1500 mg/pot may have encouraged greater use of polymer-derived carbon or intensified competition for nitrogen and oxygen, potentially weakening petroleum hydrocarbon utilization. Although PHB degradation, soil oxygen availability, and nitrogen dynamics were not directly monitored in this study, the proposed mechanisms offer a plausible explanation for the observed differences in TPH reduction between the two PHB doses. These mechanisms require further evaluation through measurements of residual PHB, inorganic nitrogen, and soil oxygen status.

Although ryegrass + PHB 500 mg/pot produced the numerically highest mean TPH reduction, ryegrass alone and PHB 500 mg/pot without ryegrass achieved reductions of 38.18% and 38.80%, respectively. Their final TPH contents did not differ significantly from that of the combined treatment (*p* = 0.6631 and 0.8774). The significant planting × amendment interaction at 150 d indicates that the effect of ryegrass depended on amendment condition (Table S5). This overall interaction does not by itself demonstrate a synergistic advantage of ryegrass combined with PHB at 500 mg/pot. In planted soil, PHB at 500 mg/pot performed better than at 1500 mg/pot, whereas the combined treatment showed only small observed mean differences relative to either component alone. Given the limited replication (*n* = 3 per treatment), the factorial results are interpreted as exploratory.

Because abiotic controls and direct hydrocarbon mineralization measurements were not included, the contributions of microbial degradation, volatilization, leaching, and changes in extractability to the observed TPH reduction were not quantified separately.

### 3.2. Distinct Effects of Low and High Molecular Weight Carbon Sources on Microbial Communities

Under the same ryegrass planting condition, ryegrass alone, ryegrass + HB 550 mg/pot, and ryegrass + PHB 1500 mg/pot represented three carbon environments: no exogenous amendment, low-molecular-weight HB, and polymeric PHB. At 150 d, community compositions with ryegrass alone and ryegrass + HB 550 mg/pot were relatively similar. Mean relative abundances of *Paracoccus* were 4.83% and 5.06%, respectively, and those of *Gordonia* were 5.83% and 8.27%. In ryegrass + PHB 1500 mg/pot, these genera declined to 2.57% and 3.56%, whereas *Penicillium* increased to 1.43%, compared with 0.56% and 0.22% in the first two treatments. These differences may reflect both amendment characteristics and the amount of carbon supplied. Because the HB and PHB treatments were not carbon-matched, the effects of carbon-source properties and carbon input cannot be fully separated in this study. Rhizosphere microbial substrate preferences contribute to community assembly, and ryegrass root metabolites can selectively attract bacteria with particular metabolic capacities [13,26].

PHB is a polymeric polyester that microorganisms must first break down into assimilable small molecules using extracellular depolymerases. Microorganisms can degrade PHB in soil, and enzymatic depolymerization can release 3-hydroxybutyrate monomers and dimers [27,28]. Pot experiments have also shown preferential microbial utilization of added PHB and consequent changes in soil carbon and nutrient turnover [19]. In the present study, ryegrass + PHB 1500 mg/pot retained relatively high concentrations of 3-hydroxybutyric acid and malic acid at 150 d, at 114.08 and 33.53 mg/kg, respectively, both significantly higher than with ryegrass alone. Mean concentrations of suberic, azelaic, and sebacic acids reached 1258.17, 968.26, and 653.93 mg/kg, respectively. This treatment also had the highest proportion of *Penicillium*. Previous studies have demonstrated PHB degradation by *Penicillium oxalicum* in soil and the capacity of fungal PHB depolymerases to recognize and hydrolyze polyesters [29,30]. Sustained PHB depolymerization may thus have created a carbon niche distinct from that in unamended soil, supporting polymer-degrading taxa and associated carbon users while leaving relatively high residual concentrations of some breakdown products and organic acids late in the experiment. One possible explanation for the relatively high residual TPH is that microorganisms preferentially utilized PHB-derived carbon and related small carbon compounds, without a corresponding improvement in TPH removal.

HB is itself a small molecule, β-hydroxybutyric acid, and can enter microbial carbon metabolism relatively rapidly without requiring extracellular polymer depolymerization. In the ryegrass + HB 550 mg/pot treatment, several organic acids declined markedly in concentration from 60 to 150 d. From 60 to 150 d, suberic acid declined from 1980.25 to 49.92 mg/kg and azelaic acid from 1000.44 to 152.28 mg/kg, with the same direction of change in all three replicates. At 150 d, malic acid, 3-hydroxybutyric acid, and all three dicarboxylic acids were lower in this treatment than with ryegrass alone. The relatively high mean relative abundances of *Paracoccus* and *Gordonia*, together with metabolic capacities previously reported for members of these genera, suggest possible roles in organic acid utilization and hydrocarbon transformation. *Paracoccus* can grow on small organic acids such as malate [31]. *Gordonia* has established alkane-utilization capacity, and its *alkB* system can participate in long-chain n-alkane oxidation [32,33]. HB addition may therefore have initially stimulated bacteria capable of rapidly utilizing small carbon compounds and subsequently enriched taxa that also transform hydrocarbons. At 150 d, the organic acid profile approached that of ryegrass alone, while the treatment retained a higher proportion of *Gordonia* and slightly lower residual TPH. Taken together, the three treatments suggest that unamended planted soil supports a baseline community shaped by the ryegrass root environment. Low-molecular-weight HB may favor rapid carbon utilization and hydrocarbon transformation, whereas polymeric PHB may create a niche characterized by depolymerization and sustained carbon supply. These proposed mechanisms may help explain the observed differences in microbial community composition and organic acid profiles. These interpretations concern amendment responses in planted soils. These community patterns and predicted functions provide a useful basis for proposing microbial mechanisms, but do not establish the metabolic activities or substrate use of individual taxa. The proposed roles require further evaluation through culture-based degradation assays, functional-gene expression measurements, or other direct metabolic evidence.

### 3.3. Bacterial and Fungal Communities Respond Differently to Carbon Source Form

HB and PHB amendments were associated with more consistent compositional differences in bacterial communities than in fungal communities. Overall differences among amendment categories were detected for bacteria in all four combinations of sampling time and planting status (q ≤ 0.0144). In unplanted soil, no amendment, HB, and PHB differed pairwise at 60 d; at 150 d, both HB and PHB also differed from no amendment. In planted soil, treatment divergence emerged later, with a significant difference between HB and PHB at 150 d. A separate overall model likewise showed that amendment type explained 21.1% of bacterial community variation at 150 d, while the amendment × planting interaction explained 19.8%. These results indicate that bacterial communities respond to carbon addition and distinguish between HB and PHB as different forms of carbon.

**Figure 6:**
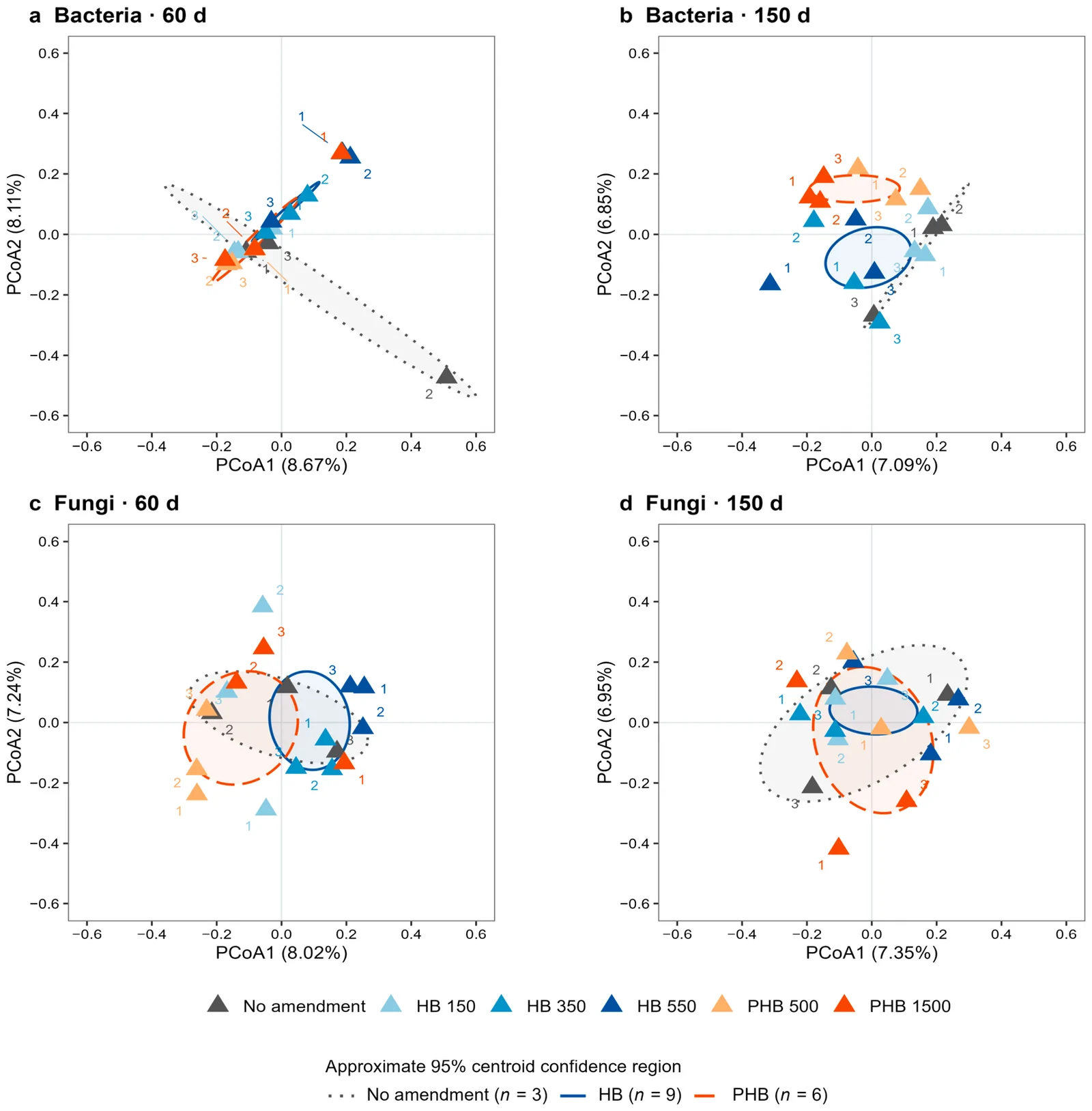
PCoA of bacterial and fungal communities in planted soil. Panels (**a**) and (**b**) show bacteria at 60 and 150 d, and panels (**c**) and (**d**) show fungi at 60 and 150 d, respectively. Point colors distinguish individual doses. Ellipses pool doses into no amendment, HB, and PHB categories and indicate approximate 95% confidence regions for group centroids. Numbers 1–3 identify the three independent pot replicates within each treatment.

**Figure 7:**
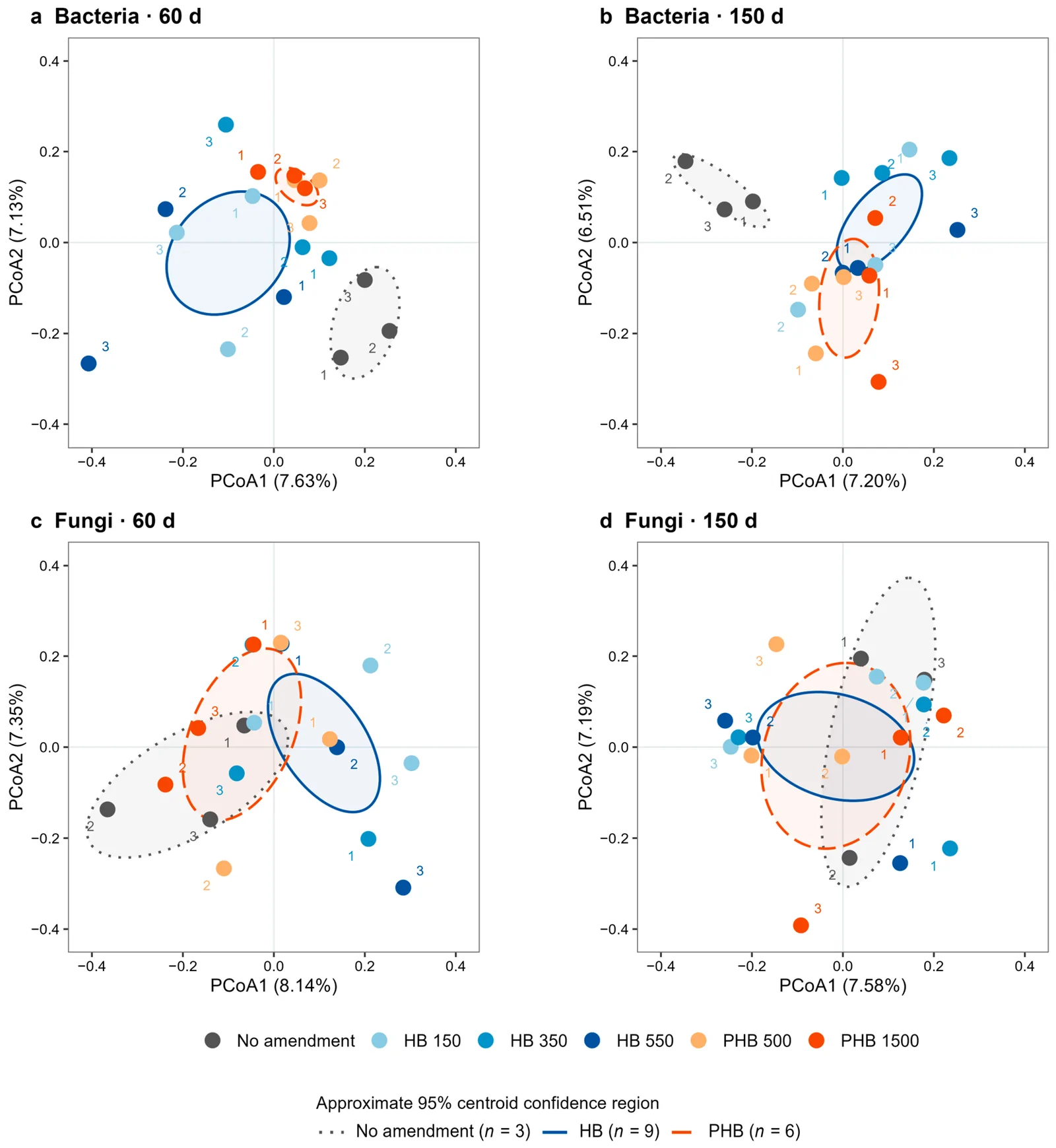
PCoA of bacterial and fungal communities in unplanted soil. Panels (**a**) and (**b**) show bacteria at 60 and 150 d, and panels (**c**) and (**d**) show fungi at 60 and 150 d, respectively. Point colors distinguish individual doses. Ellipses pool doses into no amendment, HB, and PHB categories and indicate approximate 95% confidence regions for group centroids. Ellipse overlap is used only to visualize community patterns; significance is determined by PERMANOVA. Numbers 1–3 identify the three independent pot replicates within each treatment.

Fungal communities did not show equally consistent differentiation among amendments. Their variation was associated mainly with planting, and samples receiving different amendments overlapped more extensively. In ordinations performed separately by planting status, no amendment, HB, and PHB were generally more clearly differentiated in the bacterial panels. The three approximate confidence ellipses for group centroids overlapped more in the fungal panels, particularly at 150 d (Figure 6 and Figure 7). The fungal planting effect at 150 d also depended on the distance metric, providing less consistent statistical evidence than for bacteria. Under petroleum hydrocarbon contamination, both plant presence and plant identity can strongly restructure soil bacterial and fungal communities [34]. In the present study, exogenous carbon primarily altered bacterial community composition, whereas fungal communities were more strongly influenced by plant presence and background soil variation.

This difference may reflect the resource-use strategies of bacteria and fungi. Soil bacteria include many taxa that respond rapidly to readily available carbon, and carbon sources with different chemical properties can select different bacterial assemblages [35]. Organic amendment experiments also indicate that bacteria often respond to added substrates by changing community composition, whereas fungi may respond mainly through increased abundance [36]. Some bacteria can directly utilize HB, allowing relatively rapid selection for taxa adapted to low-molecular-weight carbon. PHB utilization depends on polymer degradation and therefore creates a carbon niche distinct from that of HB. The related biodegradable polyester PHBV can form microbial activity hotspots in soil and markedly alter bacterial community composition [15]. These contrasting responses may reflect differences in the resource-use strategies of bacteria and fungi under HB and PHB amendment. Further investigation of their metabolic activities would help clarify the links between community responses, organic acid dynamics, and hydrocarbon transformation.

The target TPH loading was selected with reference to published measurements for dry oil lake soils in Kuwait [37]. However, this experiment used artificially spiked Guangzhou soil and mineral engine oil, rather than long-term field-contaminated Kuwaiti soil. Similar bulk TPH concentrations do not establish equivalence in contaminant composition, ageing, bioavailability, soil physicochemical properties, or indigenous microbial communities. Pots were maintained under a rain shelter with irrigation and fertilization, so the experiment did not reproduce the environmental conditions of Kuwaiti field sites. Because soil water-holding capacity was not measured, the role of soil water retention in microbial activity and hydrocarbon removal could not be evaluated directly. The findings should therefore be interpreted as preliminary treatment comparisons under the tested pot conditions. Their applicability to Kuwait requires validation using locally contaminated soils under relevant environmental and management conditions.

Because leaching from the pots was not controlled, possible hydrocarbon losses through this pathway cannot be excluded. The observed decline in soil TPH should therefore not be interpreted as a direct measure of biodegradation.

## 4. Materials and Methods

### 4.1. Soil and Experimental Materials

Soil was collected in Guangzhou, China. Stones and roots were removed, after which the soil was air-dried, passed through a 2.0 mm sieve, and thoroughly mixed. Before engine-oil spiking, the background soil had a pH of 7.05 and a TPH content of 0.007%. Complete physicochemical properties of the background soil are provided in Table S1. Soil particle-size distribution was determined using a Mastersizer 3000E laser particle size analyzer (Malvern Panalytical Ltd., Malvern, UK). The silt and clay fractions were 5.44% and 3.58%, respectively, and the sand fraction was calculated by difference as 90.98% (Table S1). Soil water-holding capacity was not measured. The contaminant was SJ10W40 mineral engine oil, and the test plant was perennial ryegrass (*Lolium perenne* L.). The amendments were β-hydroxybutyric acid (HB; purity 80%) and polyhydroxybutyrate (PHB; purity 98%; powder passed through an 800-mesh sieve).

### 4.2. Pot Experiment and Treatment Design

The pot experiment was conducted from 13 December 2025 to 18 May 2026. Two planting levels, with and without ryegrass, were fully crossed with six amendment conditions to yield 12 treatments (Table 1). Each treatment comprised three independent pots, giving 36 pots in total. HB and PHB doses were defined by the actual mass of material applied. Amendment-derived carbon inputs were estimated by multiplying the applied material mass by its purity and theoretical carbon mass fraction. The calculations used purities of 80% for HB and 98% for PHB, with carbon mass fractions derived from the molecular formula of HB (C_4_H_8_O_3_) and the PHB repeat unit (C_4_H_6_O_2_). HB doses of 150, 350, and 550 mg/pot corresponded to estimated carbon inputs of 55.4, 129.2, and 203.1 mg C/pot, respectively. PHB doses of 500 and 1500 mg/pot corresponded to 273.5 and 820.4 mg C/pot, respectively.

Engine oil was added at 50 mL per 1 kg of soil and thoroughly mixed to achieve a target TPH content of approximately 3.8%. This target contamination level was selected with reference to the mean TPH concentration reported for dry oil lake soils in the Burgan oil field, Kuwait (35,740.6 mg/kg, approximately 3.574%; [37]). All pots received soil from a single batch that had been thoroughly homogenized after engine-oil spiking. The common day-0 soil sample was collected from this batch before HB or PHB addition. The day-0 mean TPH concentration was used as the initial reference for all treatments. Square pots measuring 12 × 11 × 13 cm were fitted with geotextile liners and trays. Each pot initially contained 1 kg of soil. Each pot was prewetted with 300 mL of deionized water and equilibrated for 1 week.

Seeds were soaked for 3–4 h, empty seed husks were removed, and the seeds were germinated for 3 d in moist seedling trays. Twenty clusters of three seedlings each were transplanted into each planted pot, giving 60 seedlings per pot. All transplanted seedlings survived to the final harvest, with no recorded mortality or replanting. Plant height was not recorded. Pots were randomly arranged under a 3 × 3 × 2.1 m rain shelter open on all four sides and repositioned regularly. During cultivation, 50 mL of deionized water was added every 2 d. The application schedules for the nutrient solution and solid fertilizers are provided in Table S2. Solid fertilizers were applied as aqueous solutions. The solid-fertilizer doses were 135.75 mg of urea and 50.00 mg of potassium chloride per pot per application on days 90 and 120, and 74.00 mg of calcium dihydrogen phosphate monohydrate per pot on day 90. These doses corresponded to 62.445 mg N, 30.000 mg K_2_O, and 41.684 mg P_2_O_5_ per pot per application, respectively. Ambient temperature and leaching from the pots were not controlled during cultivation.

### 4.3. Soil Sampling

The same 36 pots were sampled at 60 and 150 d. Each pot was an independent experimental unit, and pot identifiers were retained across sampling times and measurements. At 60 d, soil was collected at six evenly distributed locations per pot, from the surface to a depth of approximately 10 cm, and combined into an approximately 25 g composite sample. Sampling holes were backfilled with adjacent soil and watered. At 150 d, shoots were cut at the soil surface and roots were removed before all remaining soil in each pot was thoroughly mixed. All remaining soil in unplanted pots was mixed in the same manner. Approximately 900 g of soil remained per pot. Shoot and root dry biomass were measured at 150 d, and total dry biomass was calculated for each pot as their sum. The measurement procedure and descriptive results are provided in Supplementary Method S4 and Table S4, respectively.

Sampling at 60 and 150 d took 4 and 8 h, respectively. Soil samples were placed in sterile sealed bags and held at −18 °C during sampling. They were transported on dry ice for approximately 1 h and subsequently stored at −80 °C. The interval between sampling and analysis was 4 d.

TPH and C10–C40 hydrocarbons were measured in all 12 treatments, and all treatments were analyzed by bacterial and fungal amplicon sequencing. Organic acids were measured only in the six planted treatments, yielding 18 samples per time point and 36 samples in total from 18 independent pots. Moist soil was used for bacterial community and organic acid analyses. Organic acids were not measured in unplanted treatments, and the corresponding observations were treated as missing rather than replaced with zeros.

### 4.4. Chemical Analyses

TPH was determined gravimetrically as n-hexane-extractable material using the extraction–gravimetry procedure of USEPA Method 9071B [38]. Soil samples were dehydrated with anhydrous sodium sulfate and subjected to Soxhlet extraction with n-hexane. The extracts were collected in preweighed flasks, and the solvent was removed by rotary evaporation. After cooling in a desiccator, the flasks were weighed, and the residue mass was calculated as the increase relative to the flask tare mass. TPH was calculated as the residue mass divided by the dry soil mass and expressed as a percentage.

C10–C40 hydrocarbons were analyzed by an external testing laboratory according to HJ 1021–2019 (Soil and sediment—Determination of petroleum hydrocarbons (C10–C40)—Gas chromatography; [39]). Soil samples were extracted with n-hexane/acetone and analyzed by gas chromatography with flame ionization detection (GC–FID). Hydrocarbons within the retention-time window from n-decane (C10) to n-tetracontane (C40) were quantified by external calibration. Analytical quality control included blanks, duplicate samples, and matrix-spike recovery samples. Results were expressed as percentages of dry soil mass (%).

TPH reduction was used to evaluate remediation performance, while C10–C40 hydrocarbons were used to characterize compositional changes. Treatments at the same sampling time were compared using individual pot measurements. The descriptive percentage reduction in TPH was calculated as the difference between the common day-0 mean and the treatment mean at the relevant sampling time, divided by the day-0 mean and multiplied by 100. The common day-0 sample did not constitute an independent paired baseline for each pot. Reductions in C10–C40 relative to day 0 were not calculated. Absolute differences in mass percentages were expressed in percentage points.

Soil samples were submitted to Shanghai Personalbio Technology Co., Ltd. (Shanghai, China) for targeted analysis of 65 organic acid assay entries using ultra-performance liquid chromatography coupled with electrospray ionization tandem mass spectrometry (UPLC–ESI–MS/MS; ExionLC AD–QTRAP 6500+, AB SCIEX, Framingham, MA, USA) in multiple reaction monitoring (MRM) mode. Preparation of the analytical aliquots and instrumental conditions are described in Supplementary Method S5. Data acquisition and quantitative processing used Analyst 1.6 and MultiQuant 3.0.3 (AB SCIEX, Framingham, MA, USA), respectively.

### 4.5. Microbial Community and Predicted Functional Analyses

DNA was extracted from 0.25 g soil subsamples using the DNeasy PowerSoil kit (QIAGEN GmbH, Hilden, Germany), as described by Mo et al. [40]. At both sampling times, the 16S rRNA gene V4 region and fungal ITS2 region were amplified using primer pairs 515F/806R and ITS3/ITS4, respectively, and sequenced on an Illumina NovaSeq platform (Illumina, Inc., San Diego, CA, USA) using paired-end 250-bp sequencing. Sequence quality control used fastp v0.14.1, and amplicon sequence variants (ASVs) were generated using DADA2 in QIIME 2. Taxonomy was assigned using a naive Bayes classifier with a confidence threshold of 0.8, against SILVA v138 for 16S and UNITE v8.0 for ITS2. For each marker, the same microbial analysis methods and software versions were used at 60 and 150 d. The 16S and ITS2 datasets were processed using QIIME 2 framework 2020.11.0.dev0+4.g250e9bb and q2-dada2 plugin 2020.11.0.dev0. Primer sequences and the documented processing settings are provided in Supplementary Method S2.

Bacterial 16S rRNA gene and fungal ITS amplicon sequence variant (ASV) count tables and taxonomic annotations supplied by the sequencing provider were used. Bacterial composition was aggregated by complete genus-level taxonomic lineage. Relative abundances were calculated using all 16S ASV reads in each sample as the denominator, and the 10 taxonomically resolved genera with the highest mean abundances across both sampling times were displayed.

Treatment effects on community composition were evaluated separately at 60 and 150 d using complete ASV abundance matrices, rather than matrices aggregated by taxonomic labels. Principal coordinates analysis (PCoA), based on Bray–Curtis distances after Wisconsin standardization with the Lingoes correction applied, was used to visualize community structure. PERMANOVA tested the effects of planting, the six-level amendment factor, and their interaction using Euclidean distances calculated from Hellinger-transformed abundance data. The same effects were also tested using Bray–Curtis distances calculated from relative abundances as a sensitivity analysis. Within each sampling time and planting status, separate PERMANOVA models compared no amendment, pooled HB doses, and pooled PHB doses. Homogeneity of multivariate dispersion was also assessed for each comparison family. ANCOM-BC2 was used for genus-level comparisons of each dose with its corresponding unamended control. Only taxonomically resolved genera that passed both multiple-testing correction and the pseudocount sensitivity check were retained. Preprocessing and model settings are described in Supplementary Method S2. Cross-time changes in fungal genera were not inferred because identical representative sequences received inconsistent taxonomic annotations in the two datasets. Fungal genus-level reporting was restricted to the provider-annotated relative abundance of *Penicillium* in planted treatments at 150 d.

Bacterial KEGG Orthology (KO) functions were predicted using PICRUSt2 and normalized to a total of one million units per sample (CPM). Seven KOs associated with hydrocarbon transformation were selected. After log_2_(CPM + 1) transformation, limma was used to compare each of the five amendment treatments with the unamended control within each sampling time and planting status. Stratified predictions at 60 d and taxonomic annotations were used to calculate the proportion of each KO’s total predicted abundance attributable to each genus in the same sample. The KO list and calculation details are provided in Supplementary Method S3. These results represent predicted functional potential, rather than measured gene abundances or actual contributions to hydrocarbon degradation.

### 4.6. Statistical Analysis

Statistical analyses were conducted in DMSAS 2.0 and R 4.4.3 (R Foundation for Statistical Computing, Vienna, Austria), primarily using vegan 2.7.2, ANCOMBC 2.8.1, and limma 3.62.2. Data are presented as mean ± standard deviation (SD; *n* = 3), and treatments were compared separately at each sampling time. Measured variables were assessed using Levene’s test. One-way analysis of variance followed by Duncan’s multiple range test was used when variances were homogeneous, whereas Games–Howell comparisons were used when variances were unequal. Overall treatment differences in suberic, azelaic, and sebacic acids at 150 d were assessed using Welch’s test. A factorial analysis was performed separately for residual TPH concentrations at 60 and 150 d to evaluate the effects of planting, amendment condition, and their interaction. Mean differences in residual TPH and their 95% confidence intervals were estimated from the same factorial model for the two principal treatment comparisons at 150 d.

Permutation tests used 9999 permutations. For microbial analyses, multiple testing was controlled using the Benjamini–Hochberg (BH) procedure within prespecified comparison families. The 140 prespecified KO comparisons, comprising 7 KOs × 5 amendment treatments × 2 sampling times × 2 planting conditions, were corrected jointly as a separate testing family. The significance criteria were *p* < 0.05 for measured variables and q < 0.05 for BH-adjusted results. Between-group inferences were treated as exploratory evidence. Changes from 60 to 150 d were summarized descriptively using within-pot differences. Additional correction and plotting procedures are described in Supplementary Methods S2 and S3.

## 5. Conclusions

This fully crossed pot experiment combining ryegrass planting with different HB and PHB doses compared treatment effects on TPH removal, organic acid dynamics, and microbial communities in engine-oil-contaminated soil. The observed treatment differences may reflect amendment dose and characteristics, as well as plant presence.

Over the 150 d remediation period, ryegrass combined with PHB at 500 mg/pot produced the numerically highest mean TPH reduction (39.09%), exceeding the unplanted, unamended control by 18.91 percentage points. However, final TPH concentrations did not differ significantly from those with ryegrass alone or PHB alone at the same dose (500 mg/pot). Increasing the PHB dose to 1500 mg/pot significantly increased final residual TPH in planted soil, demonstrating that greater carbon input from an exogenous polymer does not necessarily improve petroleum hydrocarbon removal.

Among the six planted treatments, HB and PHB produced distinct patterns of organic acid change and microbial community responses. With HB at 550 mg/pot, suberic and azelaic acids declined markedly during the later remediation period, accompanied by relatively high abundances of *Gordonia* and *Paracoccus*. PHB at 1500 mg/pot was associated with late accumulation of some organic acids and a relatively high abundance of *Penicillium*. One possible explanation for these patterns is that HB may favor relatively rapid microbial carbon utilization, whereas PHB may provide a more sustained carbon supply through gradual depolymerization. Across the full experiment, community analyses further showed more consistent amendment-associated compositional differences in bacterial than in fungal communities and indicated that plant presence modified bacterial responses to exogenous carbon.

Moderate PHB addition may be a candidate strategy for regulating plant-assisted remediation of petroleum-contaminated soils, but a higher polymer dose provided no further remediation benefit. Given the limited biological replication (*n* = 3 independent pots per treatment), ecological conclusions drawn from the microbial community analyses should be interpreted with caution. The use of freshly engine-oil-contaminated Guangzhou soil limits direct extrapolation to long-weathered petroleum-contaminated soils in Kuwait. Future work should combine experiments that use locally contaminated Kuwaiti soils under relevant environmental and management conditions with measurements of polymer degradation dynamics and direct assays of hydrocarbon-degradation functions. Such studies are needed to clarify the relationship between exogenous carbon utilization and petroleum hydrocarbon degradation and to guide optimization of plant–microbe remediation strategies.

## Figures and Tables

**Table 1:** Treatments in the pot experiment.

Amendment Condition	Without Ryegrass	With Ryegrass
No amendment	CK	T1
HB 150 mg/pot	T2	T3
HB 350 mg/pot	T4	T5
HB 550 mg/pot	T6	T7
PHB 500 mg/pot	T8	T9
PHB 1500 mg/pot	T10	T11

## Data Availability

The raw amplicon sequencing reads and associated sample metadata are available from the corresponding authors upon reasonable request.

## Supplementary Materials

The following supporting information can be downloaded at: https://www.mdpi.com/article/10.3390/molecules31213825/s1. Supplementary Method S1. Soil properties and fertilization records; Supplementary Method S2. Amplicon sequencing and community analysis settings; Supplementary Method S3. Functional analysis and plotting settings; Supplementary Method S4. Biomass measurement; Supplementary Method S5. Targeted organic acid analysis; Table S1. Background physicochemical properties of the experimental soil before engine-oil spiking; Table S2. Nutrient solution application records and solid-fertilizer doses per pot; Table S3. Mean relative abundances of focal genera in planted soil at 150 d (%); Table S4. Ryegrass dry biomass at 150 d; Table S5. Holm-adjusted P values for the effects of planting, amendment condition, and their interaction on residual TPH concentrations; Table S6. Model-based mean differences in residual TPH and 95% confidence intervals for the principal treatment comparisons at 150 d; Figure S1. Relative variation in 65 organic acid assay entries across 36 samples from six ryegrass-planted treatments at 60 and 150 d (three pot replicates per treatment at each time); Figure S2. Responses of bacterial genera that passed joint BH correction and the pseudocount sensitivity check for zero handling; Figure S3. Overview of predicted functions associated with petroleum hydrocarbon degradation.
