# Supplementary material for: Effects of Ryegrass and Exogenous Carbon Addition on Petroleum Hydrocarbon Removal and Soil Microbial Communities
Source: Molecules. 2026 Oct 23;31(21):3825. doi: 10.3390/molecules31213825 (PMC13765323; doi:10.3390/molecules31213825)
Supplement: Supplementary file 1 [file molecules-31-03825-s001.zip › molecules-4649310-supplementary.pdf]

Supplementary material

Effects of ryegrass and exogenous carbon addition on petroleum hydrocarbon removal and soil microbial communities

Supplementary Method S1. Soil properties and fertilization records

Table S1. Background physicochemical properties of the experimental soil before engine-oil spiking

Property	Value	Unit	Property	Value	Unit
TPH	0.007	%	TK	12.884	g/kg
pH	7.05	Dimensionless	NH_4	23.919	mg/kg
Organic matter	25.835	g/kg	NO_3	1.856	mg/kg
TN	0.846	g/kg	Available P	4.717	mg/kg
TP	0.199	g/kg	Readily available K	144.724	mg/kg
Sand	90.98	%	Silt	5.44	%
Clay	3.58	%			

The sand fraction was calculated as 100% minus the silt and clay fractions.

Table S2. Nutrient solution application records and solid-fertilizer doses per pot.

Nutrient solution

Material	Application time (d)	Quantity	Nutrient content	Equivalent nutrient application per occasion (kg/ha)
Nutrient solution	1, 15, 30, 45, 60, 75, 90	25 mL	N 8 g/L<br>P_2O_5 16 g/L<br>K_2O 6 g/L	N 12.01<br>P_2O_5 24.01<br>K_2O 9.00

The nutrient-solution quantity applies to each listed application time.

Solid fertilizers

Fertilizer	Application time (d)	Preparation quantity (g per 2 L)	Fertilizer dose (mg pot^{-1} application^{-1})	Nutrient input (mg pot^{-1} application^{-1})
Urea	90, 120	5.43	135.75	N: 62.445
Potassium chloride	90, 120	2.00	50.00	K_2O: 30.000

Fertilizer	Application time (d)	Preparation quantity (g per 2 L)	Fertilizer dose (mg pot^{-1} application^{-1})	Nutrient input (mg pot^{-1} application^{-1})
Calcium dihydrogen phosphate monohydrate	90	2.96	74.00	P_2O_5: 41.684

For solid fertilizers, preparation quantities refer to the amounts used to prepare the fertilizer solutions, not the amounts applied to individual pots. Per-pot doses were calculated using a preparation volume of 2 L and an application aliquot of 50 mL. The doses listed apply to each stated application time. Nutrient inputs were calculated using contents of 46% N in urea, 60% K_2O in potassium chloride, and 56.33% P_2O_5 in calcium dihydrogen phosphate monohydrate. Phosphorus and potassium inputs are expressed as P_2O_5 and K_2O equivalents, respectively.

Supplementary Method S2. Amplicon sequencing and community analysis settings

DNA was extracted from 0.25 g soil subsamples using the DNeasy PowerSoil kit (QIAGEN GmbH, Hilden, Germany). For each marker, 36 libraries were analyzed at each sampling time, representing three independent pots per treatment. The same pots were sampled at 60 and 150 d. The 16S rRNA gene V4 primers were 515F (5′-GTGCCAGCMGCCGCGGTAA-3′) and 806R (5′-GGACTACHVGGGTWTCTAAT-3′). The fungal ITS2 primers were ITS3 (5′-GCATCGATGAAGAACGCAGC-3′) and ITS4 (5′-TCCTCCGCTTATTGATATGC-3′). Both sampling times used the same marker-specific primer pairs and Illumina NovaSeq PE250 sequencing (Illumina, Inc., San Diego, CA, USA).

Quality control used fastp v0.14.1. For each marker, the same processing workflow and software versions were used at 60 and 150 d. For both 16S and ITS2, quality-controlled, previously merged reads were processed using DADA2 denoise_single in QIIME 2 (framework 2020.11.0.dev0+4.g250e9bb; q2-dada2 plugin 2020.11.0.dev0). The recorded parameters were trunc_len = 0, trim_left = 0, max_ee = 2, trunc_q = 2, pooling_method = independent, chimera_method = consensus, min_fold_parent_over_abundance = 1, and n_reads_learn = 100000. Thus, paired-end assembly preceded this DADA2 step; denoise_single does not imply that sequencing was single-end. Taxonomic assignment used QIIME 2 naive Bayes classifiers with a confidence threshold of 0.8, using SILVA v138 for 16S and UNITE v8.0 for ITS2 at both sampling times.

The complete vendor-filtered 16S count tables contained 6,321 ASVs at 60 d and 10,773 at 150 d; the corresponding ITS2 tables contained 1,475 and 1,680 ASVs. These are dataset-wide feature counts, not species counts or per-pot richness. The 60-d 16S table included 33 archaeal ASVs, which were retained in the all-16S read denominator described in Section 4.5. Final ASV-table counts per library ranged from 58,287 to 82,998 for 16S at 60 d, 65,232 to 85,015 for 16S at 150 d, 63,652 to 81,735 for ITS2 at 60 d, and 67,421 to 85,703 for ITS2 at 150 d. These counts refer to retained observations in the vendor-filtered ASV tables, not raw sequencing output.

The 60-d and 150-d samples corresponded to separate sequencing deliveries, with A- and B-prefixed sample identifiers, respectively. Community treatment effects were evaluated within each sampling time using complete ASV matrices, without aggregation by taxonomic labels. Identical fungal representative sequences received inconsistent genus-level annotations across the two datasets, and the cause of this inconsistency was not established. Cross-time fungal genus changes were therefore not inferred; the reported *Penicillium* proportions at 150 d retain the provider-

supplied annotation. Use of the same overall workflow does not establish the absence of sequencing or processing batch effects, and the within-time comparisons do not resolve potential confounding between sampling time and sequencing delivery.

PCoA used Bray–Curtis distances after Wisconsin standardization and applied the Lingoes correction. For each sampling time, PERMANOVA tested the effects of planting, the six-level amendment factor, and their interaction using Euclidean distances calculated from Hellinger-transformed abundance data. Each design term was evaluated marginally while controlling for the remaining terms. Sensitivity analyses repeated the PERMANOVA using Bray–Curtis distances calculated from relative abundances. Within each sampling time and planting status, separate PERMANOVA models based on Bray–Curtis distances after Wisconsin standardization compared no amendment, pooled HB doses, and pooled PHB doses. Homogeneity of multivariate dispersion was tested for each comparison family. ANCOM-BC2 models were fitted in four subsets defined by the two sampling times and two planting conditions. All valid genus-lineage contrasts against the control across the four models were corrected jointly using the BH procedure. Omnibus, pairwise, and dispersion-test P values were corrected separately by analysis module and prespecified comparison family.

Supplementary Method S3. Functional analysis and plotting settings

Each KO was normalized to CPM using the total predicted abundance of all KOs in the same sample as the denominator. The seven KOs were K00496 (*alkB*/*alkM*), K05549 (*benA*/*xylX*), K05550 (*benB*/*xylY*), K00446 (*dmpB*/*xylE*), K07104 (*catE*), K00448 (*pcaG*), and K00449 (*pcaH*). BH correction was applied only to finite P values from estimable comparisons.

Organic acid entries retained the original values and explicit zeros in the assay reports; records with similar names were not merged. Focal compounds were selected based on amendment relevance, chemical class, and observed patterns of change. For the organic acid heatmap, values were transformed as $\log_{10}(x + 1)$, and each assay entry was standardized to a row Z score across its 36 samples. For the 33-KO heatmaps in Figure S3, treatment means were standardized by row separately at each sampling time. Genus-contribution plots displayed proportions directly. Unmeasured values and explicit zeros were retained as distinct categories.

Supplementary table

Table S3. Mean relative abundances of focal genera in planted soil at 150 d (%)

Treatment	*Paracoccus* (%)	*Gordonia* (%)	*Penicillium* (%)
Ryegrass only	4.83	5.83	0.56
Ryegrass + HB 550 mg/pot	5.06	8.27	0.22
Ryegrass + PHB 500 mg/pot	2.26	5.71	0.36
Ryegrass + PHB 1500 mg/pot	2.57	3.56	1.43

Each value is the mean of three pots and is used only to describe compositional differences under the same planting condition.

Supplementary Method S4. Biomass measurement

At 150 d, ryegrass was harvested from all 18 planted pots. Shoots were cut at the soil surface. Roots, including fine roots, were separated from the soil, rinsed with deionized water, and drained. Fresh shoot and root masses were recorded separately. The samples were placed in separate kraft paper bags and dried at 90 °C for 1 h, followed by 70 °C for 48 h. Samples were weighed using a balance with 0.1 mg readability, dried for a further 2 h at 70 °C, and weighed again. Constant mass was defined as a difference of less than 1.0 mg between successive weighings. Shoot and root dry biomass were expressed in g/pot, and total dry biomass was calculated for each pot as their sum. Values are reported as mean ± SD for three independent pots per treatment.

Table S4. Ryegrass dry biomass at 150 d

Treatment	Shoot dry biomass (g/pot)	Root dry biomass (g/pot)	Total dry biomass (g/pot)
Ryegrass only	2.010 ± 0.496	3.117 ± 0.383	5.127 ± 0.879
Ryegrass + HB 150 mg/pot	2.552 ± 0.497	4.063 ± 0.183	6.615 ± 0.602
Ryegrass + HB 350 mg/pot	2.306 ± 0.482	3.651 ± 2.079	5.956 ± 2.557
Ryegrass + HB 550 mg/pot	2.149 ± 0.510	3.247 ± 0.627	5.396 ± 1.137
Ryegrass + PHB 500 mg/pot	2.683 ± 0.195	3.550 ± 0.298	6.233 ± 0.196
Ryegrass + PHB 1500 mg/pot	2.177 ± 0.090	2.469 ± 0.230	4.646 ± 0.301

Values are mean ± SD for three independent pots per treatment at the 150 d harvest. Total dry biomass was calculated for each pot as the sum of shoot and root dry biomass. Only planted treatments are included; unplanted treatments were not assigned zero biomass. This table provides descriptive summaries without inferential significance labels.

HB, β-hydroxybutyric acid; PHB, polyhydroxybutyrate. Amendment doses are expressed as the mass applied per pot.

Table S5. Holm-adjusted P values for the effects of planting, amendment condition, and their interaction on residual TPH concentrations.

Sampling time	Planting	Amendment condition	Planting × amendment condition
60 d	0.0352	0.0056	0.1017
150 d	0.0027	0.00052	0.00062

Separate factorial models were fitted at each sampling time, with three independent pots per treatment combination. HC3 heteroscedasticity-robust tests were used to account for potential differences in variance among treatments. Main effects were averaged equally across the levels of the other factor. P values were adjusted using Holm's method across the six TPH tests shown in the table.

Table S6. Model-based mean differences in residual TPH and 95% confidence intervals for the principal treatment comparisons at 150 d.

Treatment comparison	Mean difference in residual TPH (percentage points)	95% simultaneous confidence interval
Ryegrass + PHB 500 mg/pot − ryegrass alone	−0.0343	[−0.2210, 0.1525]
Ryegrass + PHB 500 mg/pot − PHB 500 mg/pot alone	−0.0108	[−0.1834, 0.1618]

Estimates were obtained from the factorial model for residual TPH at 150 d using HC3 heteroscedasticity-robust covariance estimates. Each treatment included three independent pots. Differences were calculated as the combined treatment minus the corresponding single-component treatment; negative values indicate lower residual TPH in the combined treatment. Simultaneous 95% confidence intervals were calculated across the two contrasts using a single-step multivariate-t adjustment, with 24 residual degrees of freedom. These model-based estimates supplement the original treatment-wise comparisons.

Supplementary Method S5. Targeted organic acid analysis

Soil samples were submitted to Shanghai Personalbio Technology Co., Ltd. (Shanghai, China). The laboratory's downstream preparation procedure started with 2 mL liquid aliquots. Each aliquot was freeze-dried, reconstituted in 250 µL of 20% acetonitrile in methanol, and vortexed for 3 min. An internal-standard mixture (10 µL, 250 ng/mL) was then added. After centrifugation at 12,000 rpm for 10 min at 4 °C, 200 µL of supernatant was transferred to a new tube and centrifuged again at 12,000 rpm for 5 min at 4 °C. A 180 µL portion of the resulting supernatant was transferred for LC–MS/MS analysis.

Chromatographic separation used an ExionLC AD system (AB SCIEX, Framingham, MA, USA) with a Waters ACQUITY UPLC HSS T3 column (100 × 2.1 mm, 1.8 µm; Waters Corporation, Milford, MA, USA). Mobile phase A was water containing 0.05% formic acid, and mobile phase B was acetonitrile containing 0.05% formic acid. The gradient started at 5% B at 0 min, increased to 95% B at 8 min, remained at 95% B until 9.5 min, and returned to 5% B at 9.6 min, which was maintained until 12 min. The flow rate was 0.35 mL/min, the column temperature was 40 °C, and the injection volume was 2 µL.

Detection used a QTRAP 6500+ mass spectrometer (AB SCIEX, Framingham, MA, USA) equipped with an electrospray Turbo IonSpray interface operated in positive and negative ion modes. The source temperature was 550 °C, the ion-spray voltage was 5500 V in positive mode and −4500 V in negative mode, and the curtain gas pressure was 35 psi. MRM transitions were monitored with optimized declustering potentials and collision energies for individual analytes. Data acquisition used Analyst 1.6 software (AB SCIEX, Framingham, MA, USA).

Quantitative data processing used MultiQuant 3.0.3 (AB SCIEX, Framingham, MA, USA). Peak integration was checked against the retention times and peak shapes of authentic standards, and analyte concentrations were calculated using standard calibration curves.

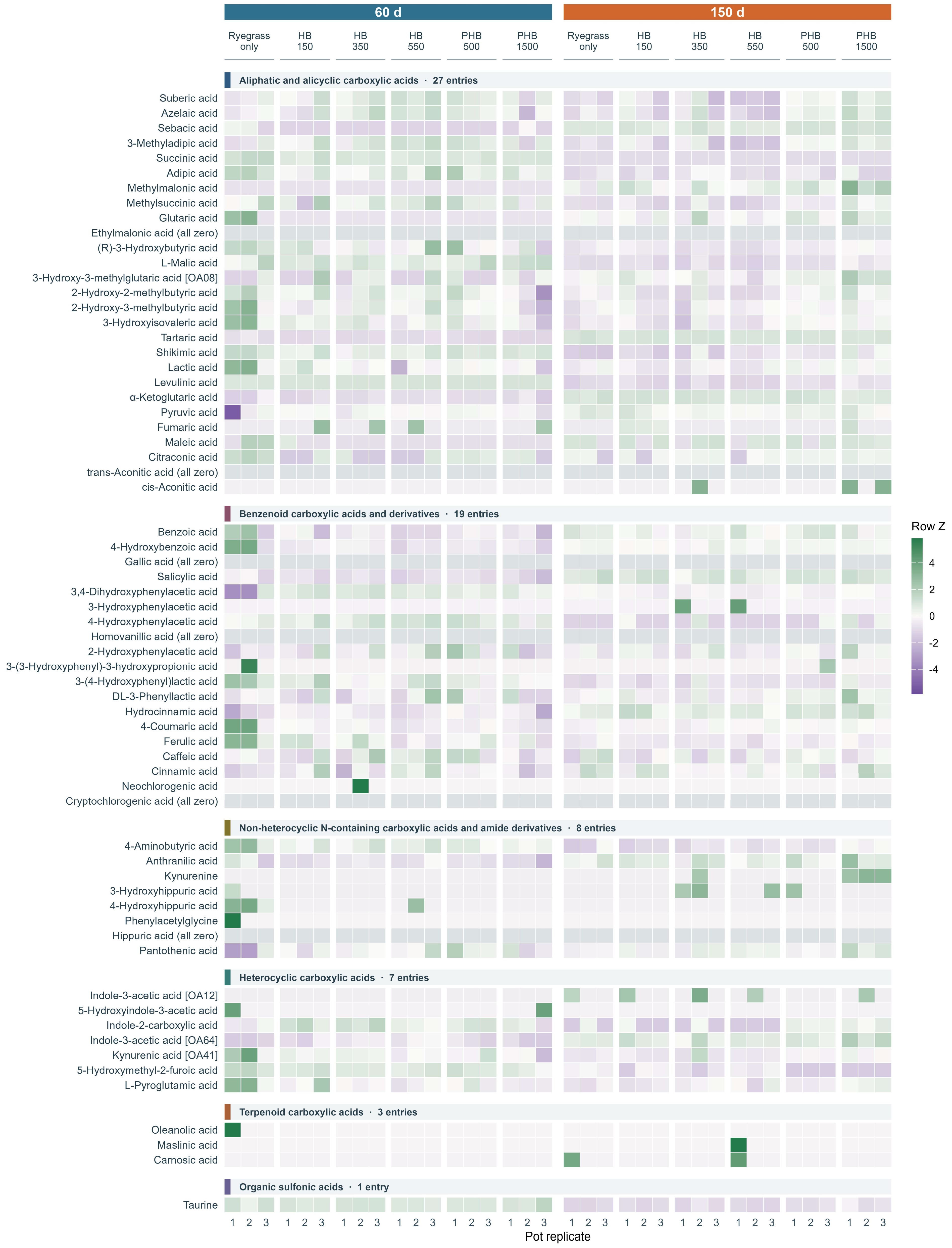

Figure S1. Relative variation in 65 organic acid assay entries across 36 samples from six ryegrass-planted treatments at 60 and 150 d (three pot replicates per treatment at each time). Rows are organized into six chemical structural classes. Colors represent row Z scores of $\log_{10}(x + 1)$-transformed concentrations, standardized across all 36 samples for each assay entry; green indicates higher relative values and purple indicates lower relative values. Entries with zero concentrations in all samples are shown in gray and labeled "all zero". Amendment doses are expressed in mg/pot. OA08 is 3-hydroxy-3-methylglutaric acid, and OA41 is kynurenic acid. The two indole-3-acetic acid assay entries are retained separately under their original IDs (OA12 and OA64).

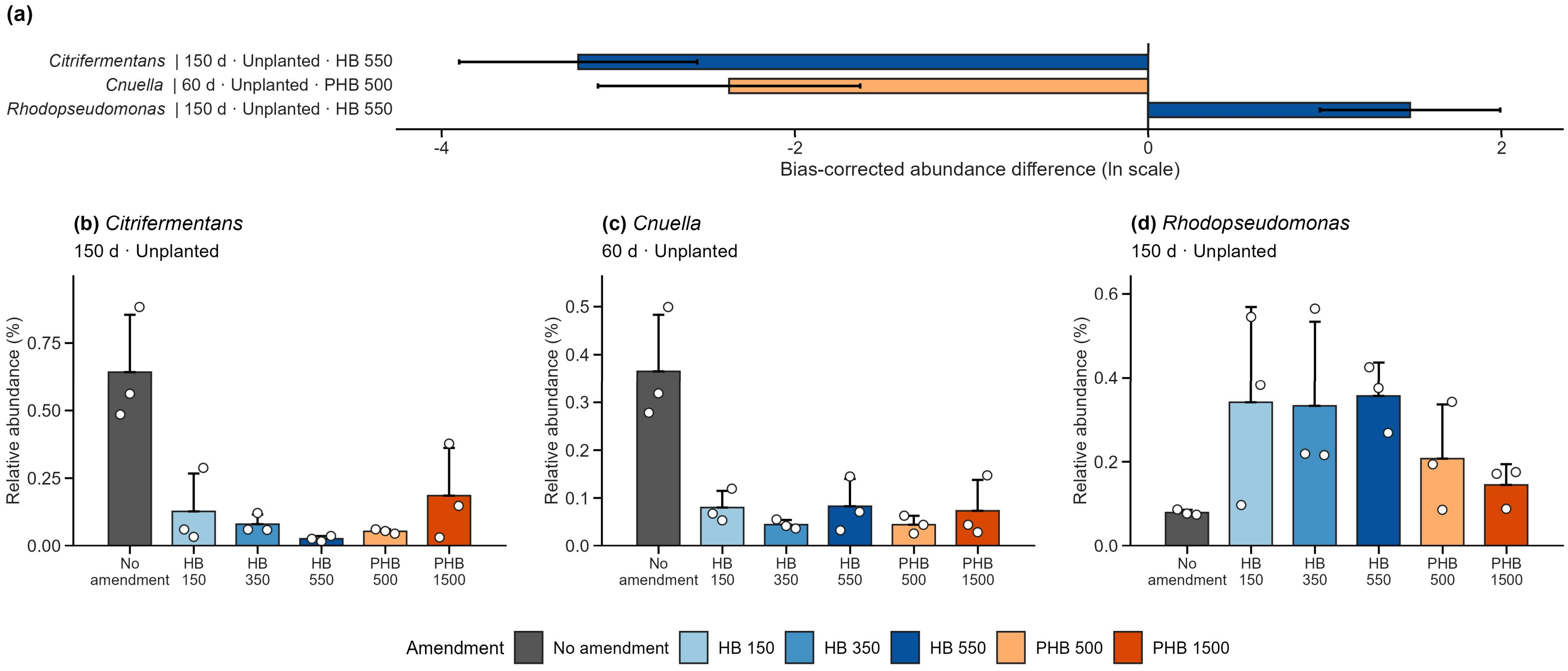

Figure S2. Responses of bacterial genera that passed joint BH correction and the pseudocount sensitivity check for zero handling. Panel a shows ANCOM-BC2 estimates of between-treatment abundance differences, with one prespecified representative comparison displayed per genus. The remaining panels show individual-pot values and means + SD for the corresponding genus across six treatments at the same sampling time and planting status. Each group contains three pots ($n = 3$). This analysis is exploratory.

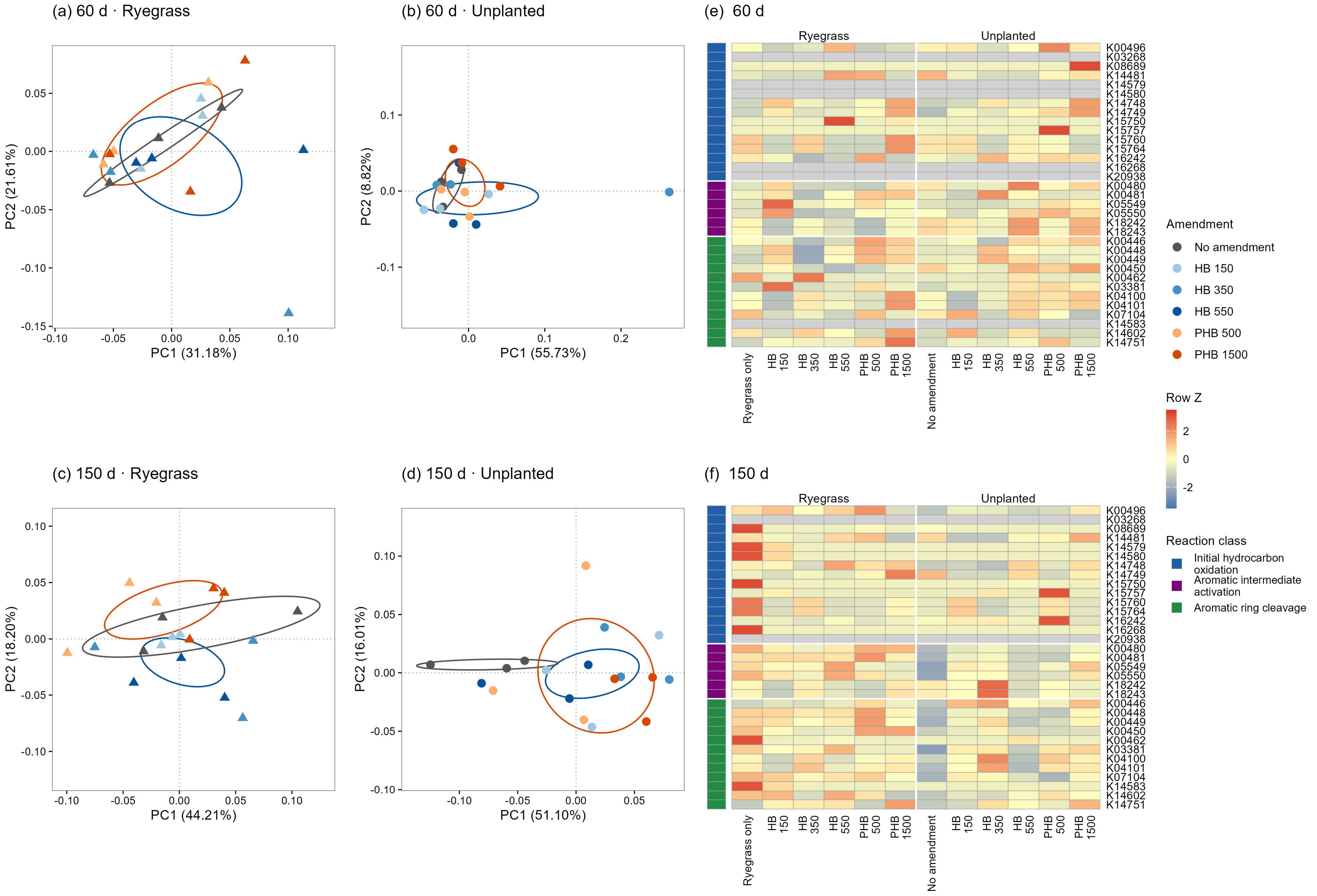

Figure S3. Overview of predicted functions associated with petroleum hydrocarbon degradation. Panels a–d show PCA of all KOs in planted soil at 60 d, unplanted soil at 60 d, planted soil at 150 d, and unplanted soil at 150 d, respectively, with 18 pots per panel. Predicted abundances were converted to within-pot proportions and Hellinger-transformed. PCA was centered without scaling by KO variance. Panels e and f show heatmaps of 33 prespecified hydrocarbon-degradation KOs at 60 and 150 d. Each cell represents the mean CPM of three pots, standardized by KO row across the 12 treatments at the same sampling time. Gray indicates that the KO was not supplied, not that its predicted abundance was zero. The two sampling times were standardized separately, so colors cannot be used to infer increases or decreases over time.
